# Nasal Carriage and Methicillin Resistance of *Staphylococcus aureus* among Schoolchildren in Sana'a City, Yemen

**DOI:** 10.1155/2021/5518317

**Published:** 2021-05-06

**Authors:** Arwa Mohammed Othman, Belques Sharaf Al-Huraibi, Rowa Mohammed Assayaghi, Huda Zaid Al-Shami

**Affiliations:** Microbiology and Immunology Department, Faculty of Medicine and Health Sciences, Sana'a University, Sana'a, Yemen

## Abstract

**Background:**

*Staphylococcus aureus* (*S. aureus*) is a frequent cause of serious health problems with high morbidity and mortality. The risk of *S. aureus* infections is increased with the emergence of methicillin-resistant *S. aureus* (MRSA). This study aims to determine the nasal carriage rate of both *S. aureus* and MRSA among schoolchildren in Sana'a city.

**Methods:**

This is a cross-sectional study conducted from January 2018 to May 2020. Five hundred and thirty-six students were enrolled. Their age ranged from 5 to 19 years with the mean age and standard deviation equal to 13.3 ± 3.5 years. Nasal swabs were collected from each student for culturing and methicillin susceptibility testing.

**Results:**

Students with positive culture were 271 (51%) males and 265 (49%) females. *S. aureus* was isolated from 129 (24%) students whereas the overall prevalence of MRSA was 8 (1.5%). *S. aureus* was significantly recovered from students at the age group of 10–14 years (*χ*^2^ = 7.02; *p*=0.03), females than males (OR = 1.96; *χ*^2^ = 10.75; *p*=0.001), and students who were admitted into hospitals (OR = 1.6; *χ*^2^ = 4.89; *p*=0.03). Nevertheless, there were no significant differences between MRSA carriage and students' age (*χ*^2^ = 2.3; *p*=0.32), gender (OR = 1.02; *χ*^2^ = 0.001; *p*=0.63), and hospital admission (OR = 1.4; *χ*^2^ = 0.25; *p*=0.62).

**Conclusions:**

The prevalence of MRSA is low among schoolchildren in Sana'a city. Age, gender, and previous hospital admission were statistically associated with nasal carriage of *S. aureus* but not MRSA nasal carriage.

## 1. Background

*Staphylococcus aureus* (*S. aureus*) is a Gram-positive, round-shaped, catalase- and coagulase-positive bacterium. It is a frequent cause of serious health problems with high morbidity and mortality. *S. aureus* colonizes the skin and nasal mucosa and thus can also be considered as normal flora. The anterior nares appear as the main reservoir site for *S. aureus* replication and spread to other body sites. Approximately 25–30% of healthy individuals are nasal carriers for *S. aureus* [1, 2]. Any damage to the epithelial barriers such as trauma and medical or surgical interventions can lead to tissue invasion by *S. aureus* [3, 4].

*S. aureus* expresses many virulence factors, and its resistance to antibiotics has gradually increased in recent years. Overuse and/or misuse of antibiotics led to the appearance of strains of *S. aureus* that are resistant to many currently used antibiotics. Penicillin-resistant *S. aureus* produces penicillinase, which hydrolyzes the *β*-lactam ring of penicillin leading to penicillin resistance. Later on, scientists developed novel semisynthetic penicillin called methicillin, which is resistant to staphylococcal *β*-lactamase [5, 6]. Methicillin successfully controlled the infection caused by penicillin-resistant *S. aureus* strains. Nevertheless, only 2 years later after methicillin use in treatment, isolation of methicillin-resistant *S. aureus* (MRSA) strains was reported. Methicillin resistance is due to the acquisition of the methicillin resistance (*mecA*) gene integrated into the chromosomal element. The *mecA* gene encodes an altered penicillin-binding protein 2a or 2′ (PBP2a or PBP2′) which permits *S. aureus* to grow in the presence of methicillin and other *β*-lactam antibiotics [7].

MRSA strains have spread worldwide and they cause numerous nosocomial outbreaks in both hospitals and communities. MRSA carriers are predisposed to skin infections, wound infections, bone and joint infections, pneumonia, septicemia, endocarditis, and occasionally toxic shock syndrome. Infections from either hospital-acquired MRSA (HA-MRSA) or community-acquired MRSA (CA-MRSA) have increased the challenge of selecting empirical antimicrobial treatments [8–10]. In healthcare institutions, HA-MRSA can be transmitted between patients, or through the hands, clothes, or equipment of healthcare workers (HCWs), and the environment [11].

Nasal carriage of MRSA among HCWs in Taiz city, Yemen, was 55.7% which is three times much greater than that among HCWs in Saudi Arabia (18%) and in Oman (15.1%)—neighboring countries [12–14]. MRSA prevalence among HCWs was 82.3% in the Gaza strip, 61.0% in Iraq, 5.3% in Iran, 4.1% in China, and 4.6% in Europe and the United States [15–19].

There is increasing evidence that CA-MRSA is spreading among healthy persons, especially children [20]. In a study conducted by AL-Haj et al., the nasal carriage rate of *S. aureus* was reported to be 23.1% among public schoolchildren in Sana'a city, but they did not test the isolated bacteria for methicillin and oxacillin sensitivity [21]. Therefore, this study aimed to determine the nasal carriage rate of both *S. aureus* and MRSA among schoolchildren in Sana'a city.

## 2. Methods

### 2.1. Study Design and Area

A cross-sectional study was conducted from January 2018 to June 2020. Six primary and secondary schools at Sana'a city were randomly chosen by a cluster sampling method. These schools included four public schools (Al Hussain Bin Ali, Roqaya, Omar Bin Al Khattab, and Khaled Bin Al Waleed) and two private schools (Al Amjad and Al Ilmiyah).

### 2.2. Study Population

Five hundred and thirty-six students were enrolled in this study. Simple random sampling was used to choose students who would participate in the study.

### 2.3. Inclusion Criteria

All randomly selected schoolchildren who agreed to participate were included.

### 2.4. Exclusion Criteria

Students who were on antibiotic therapy for one week before sample collection or those who had ulceration or pus at the nares or skin were excluded. Diabetic students were also excluded.

### 2.5. Sample Size Determination

Based on a study conducted by Al-Haj et al., who reported the prevalence of *S. aureus* among schoolchildren to be 23.1% [21], and on Central Statistical Organization at Ministry of Planning and Inter Coop (2005–2006) which stated the number of schoolchildren in Sana'a city to be about 2240000 students, the calculated sample size was 471 students at a confidence level of 99%. However, because many students liked to participate, 536 students were enrolled in this study.

### 2.6. Specimen Collection and Examinations

Samples were obtained from students by using sterile dry-cotton swabs from anterior nares. The swab was inserted 2-3 centimeters in the nasal cavity and rotated 4-5 times both clockwise and anticlockwise before swab withdrawal. Samples were labeled and transported in Amies transport media to the microbiology laboratory at the Faculty of Medicine and Health Sciences within 5-6 hours. At the microbiology laboratory, nasal swabs were inoculated on mannitol salt agar (HiMedia, India). The inoculated agar plates were incubated at 35–37°C for 24–48 h. After incubation, plates were investigated for mannitol-fermenter colonies which appear as yellow colonies on mannitol salt agar. Yellow colonies were subcultured on nutrient agar. Golden-yellow colonies on nutrient agar were further examined by Gram stain, catalase, and coagulase tests. Gram-positive cocci, arranged in grape-like clusters, catalase, and coagulase-positive were recorded as *S. aureus* [22, 23].

### 2.7. Antibiotic Susceptibility Test

All colonies confirmed to be *S. aureus* were tested for methicillin (5 *μ*g) and oxacillin (1 *μ*g) susceptibility by the modified Kirby–Bauer disc diffusion method. Using a sterile loop, colonies from nutrient agar which were confirmed to be *S. aureus* were picked up, suspended in sterile saline, and mixed to even turbidity. The turbidity intensity of bacterial suspension was adjusted in comparison with the 0.5 McFarland turbidity standard by adding saline or more bacteria. A sterile cotton swab was dipped into the bacterial suspension. Then, the Mueller-Hinton agar plate (HiMedia, India) was inoculated by swabbing in three directions to evenly distribute the inoculum and make sure there were no gaps between streaks. 5 *μ*g methicillin disc and 1 *μ*g oxacillin (HiMedia, India) were applied using a sterile needle to come in contact with the agar surface. Inoculated Muller-Hinton plates were incubated at 35–37°C for 24 h [24]. Resulted inhibition zones were measured using a ruler. Inhibition zone less than or equal to 9 mm indicated *S. aureus* to be MRSA, while inhibition zones less than or equal to 17 mm indicated oxacillin-resistant *S. aureus* (Zone Size Interpretative Chart, HiMedia).

### 2.8. Statistical Analysis

Data analysis was done using SPSS program version 20 (SPSS Inc., Chicago, IL, USA). Descriptive measures (mean ± standard deviation) were used for quantitative variables. Frequencies and percentages were used to present qualitative variables. Chi-square (*χ*^2^) test was used for verifying the existence of associations. Probability (*p*) values ≤ 0.05 were considered statistically significant.

## 3. Results

Five hundred and thirty-six nasal swabs yielded bacterial growth. The age of the 536 students ranging from 5 to 19 years with the mean age and standard deviation equal to 13.3 ± 3.5 years. They were divided into three age groups: the first age group was from 5 to 9 years, the second age group was from 10 to 14 years, and the third group was from 15 to 19 years. Out of 536, 271 (51%) students were males and 265 (49%) students were females. The number of students per class ranged from 3 to 159 students with the mean and standard deviation equal to 71 ± 34 students. Four hundred (75%) students studied at public schools while 136 (25%) studied at private schools (Table 1).

*S. aureus* was isolated from the anterior nares of 129 (24%) students. It was recovered more frequently from students at the age group of 10–14 years (63, 30%) than from students at the age group of 5–9 years (22, 22%) and students at the age group of 15–19 years (44, 19%). This difference was statistically significant (*χ*^2^ = 7.02; *p*=0.03). *S. aureus* was commonly isolated from females (80, 30%) than males (49, 18%). The gender difference was statistically significant (*χ*^2^ = 10.75; *p*=0.001). *S. aureus* was recovered from 48 (30%) students who were admitted into hospitals for either medical treatment or surgery whereas *S. aureus* was found among 81 (21%) students who reported no hospital admission. This difference was statistically significant (*χ*^2^ = 4.89; *p*=0.03). Twenty-eight (21%) students who were *S. aureus* carries had family members who worked at hospitals or any other health centers compared to 101 (25%) students who had no family members worked in hospitals or health centers. This difference was statistically nonsignificant (*χ*^2^ = 1.1; *p*=0.03) (Table 2).

Out of 129 isolated *S. aureus*, 8 (6.2%) were resistant to both methicillin and oxacillin discs with an overall prevalence of 1.5% among school students.  Table 3 shows that 3 (1.4%) students in the 10- to 14-year age group and 5 (2.2%) students in the 15- to 19-year age group but no students in the 5- to 9-year age group were carriers for MRSA. This age difference was statistically nonsignificant (*χ*^2^ = 2.3; *p*=0.32). MRSA was equally isolated from both males and females (4, 1.5%) with no significant difference (*χ*^2^ = 0.001; *p*=0.63). MRSA was isolated from 3 (1.4%) students who were admitted to hospitals while it was recovered from 5 (2.2%) who were not admitted to hospitals. This difference was statistically nonsignificant (*χ*^2^ = 2.25; *p*=0.62). Seven (1.7%) students who were MRSA carriers had no family members work at hospitals or health centers, but 1 (0.7%) student who had MRSA at his nares reported a family member works at a health center. This difference was statistically nonsignificant (*χ*^2^ = 0.69; *p*=0.41) (Table 3).

## 4. Discussion

Nasal carriage of *S. aureus* is approximately 20–30% of healthy individuals with high permanent colonization among children [25, 26]. The emergence of MRSA infections has become a worrying problem in the clinical field because MRSA strains are resistant to many antibiotics particularly *β*-lactam classes [27]. The purpose of this study was to estimate the nasal carriage rate and methicillin resistance of *S. aureus* among schoolchildren in Sana'a city.

In the present study, the prevalence of *S. aureus* among primary and secondary schoolchildren was 24% (129/536). This finding is consistent with a study conducted by Al-Haj et al., who found the colonization rate of *S. aureus* among public schoolchildren in Sana'a city to be 23.1% [21]. Our finding was also similar to those reported from other countries [28–30]. However, the nasal carriage rate of *S. aureus* in this study tends to be lower than that reported from Nigeria (56.3%), India (46.67%), the United States (39.6%), the Netherlands (36%), and Nepal (31%) [31–35]. On the other hand, the prevalence of *S. aureus* among schoolchildren in our study was higher than that reported from China (5.1%), Serbia (2.59%), and Iraq (17.75%) [36–38]. Variation in the *S. aureus* nasal carriage from one country to another might be attributed to differences in geographical distribution, sampling, culturing, and diagnostic techniques used by the researchers.

The prevalence of MRSA among schoolchildren in our study was 1.5% (8/536) which was half lower (3%) than that found by Shetty et al., in Deralakatte, India, who used oxacillin to detect MRAS among schoolchildren [39]. Moreover, the prevalence of MRSA in this study was much lower than that reported by a study performed in southwest Ethiopia (18.8%) in which the researchers used cefoxitin (30 *μ*g) discs to detect MRSA nasal carriage [40].

Comparing the prevalence of *S. aureus* in our study to the prevalence of *S. aureus* among hospitalized patients at ICU reported by Abdelmonem et al., the prevalence of *S. aureus* among schoolchildren was much less than that among hospitalized patients [12]. In addition, the prevalence of CA-MRSA among schoolchildren in our study was less than the prevalence of HA-MRSA among ICU patients (86.2% and 100%) found by Goudarzi et al. and Eftekhar et al., respectively [41, 42]. This reflects the higher prevalence of HA-MRSA than CA-MRSA.

The current study showed the nasal carriage rate of *S. aureus* in the 5- to 9-year age group to be 22% which increased significantly to 30% in the 10- to 14-year age group and then decreased to 19% in the 15- to 19-year age group. Our finding is in agreement with a study conducted by Esposito et al., who reported nasal carriage of *S. aureus* decreases while oropharyngeal carriage increases with age [43]. Decreased nasal carriage in older students may be attributed to their well-developed and stronger immune system.

Concerning gender, our study revealed a significantly higher prevalence of *S. aureus* among females than males but no difference between nasal carriage of MRSA among females and males. Al-Haj et al. reported a higher nasal carriage among females than males, but the difference was nonsignificant [21]. Moreover, Okwu et al. found nasal carriage of *S. aureus* and MRSA to be nonsignificantly higher among females than males [44]. Nevertheless, many studies conducted on schoolchildren found the frequency of *S. aureus* and MRSA nasal carriage to be nonsignificantly higher in males than in females [35, 42, 45]. Tigabu et al. described a higher prevalence of *S. aureus* among males than females but a slightly higher MRSA prevalence among females than males [30]. Statistically significant nasal colonization of *S. aureus* among females than males in our study might be due to wearing a veil among females as an obligatory custom. Wearing a veil might make the anterior nares environment warmer and more humid which in turn may favor *S. aureus* colonization.

Regarding hospitalization, this study showed a highly significant association between nasal colonization of *S. aureus* with hospitalization; however, nasal carriage of MRSA among schoolchildren showed a nonsignificant association with hospitalization. Nevertheless, no association was found between the presence of first-degree relatives who worked in hospitals or health centers and nasal colonization with *S. aureus* and MRSA. Our study is in disagreement with the study conducted in Argentina by Gardella et al., who reported no statistical difference between nasal carriage of *S. aureus* and hospitalization [46]. However, our study is consistent with a study performed in Ethiopia by Reta et al., who reported no association between MRSA prevalence and hospitalization [45]. This finding might imply *S. aureus* acquisition during hospital admission. On the other hand, the low frequency of isolated MRSA makes it difficult to conclude whether MRSA is HA-MRSA or CA-MRSA.

## 5. Conclusions

In conclusion, the prevalence of MRSA among schoolchildren in Sana'a city is low. Age, gender, and previous hospital admission were statistically associated with nasal carriage of *S. aureus* but not with MRSA nasal carriage.

## Figures and Tables

**Table 1:** Sociodemographic features of schoolchildren in Sana'a city, Yemen.

	No	%
*Age groups of students*		
5–9 years	98	18
10–14 years	210	39
15–19 years	228	43
Mean age ± SD^∗^	13.3 ± 3.5	

*Gender*		
Males	271	51
Females	265	49

*Number of students*		
Public schools	400	75
Private schools	136	25
Mean per class ± SD	71 ± 34	

^∗^SD: standard deviation.

**Table 2:** Risk factors for nasal carriage of *S. aureus* among schoolchildren in Sana'a city, Yemen.

	*S. aureus* carriers		Noncarriers		*χ* ^2^	*p*
	No.	%	No.	%
*Age groups (years)*						
5–9	22	22	76	78	7.02	0.03
10–14	63	30	147	70		
15–19	44	19	184	81		

*Gender*						
Males	49	18	222	82	10.75	0.001
Females	80	30	185	70		

*History of hospitalization*						
Yes	48	30	110	70	4.89	0.03
No	81	21	297	79		

*A family member works at hospitals or health centers*						
Yes	28	21	101	25	1.09	0.3
No	107	79	300	75		

**Table 3:** Risk factors for nasal carriage of MRSA among schoolchildren in Sana'a city, Yemen.

	MRSA^∗^ (meth + oxa)^∗^^∗^		Other bacteria		*χ* ^2^	*p*
	No.	%	No.	%		
*Age groups (years)*						
5–9	0	0	98	100	2.25	0.32
10–14	3	1.4	207	98.6		
15–19	5	2.2	223	97.8		

*Gender*						
Males	4	1.5	267	98.5	0.001	0.63
Females	4	1.5	261	98.5		

*History of hospitalization*						
Yes	3	1.9	155	98.1	0.25	0.63
No	5	1.3	373	98.7		

*A family member works at hospitals or health centers*						
Yes	1	0.7	134	99.3	0.69	0.41
No	7	1.7	394	98.3		

^∗^MRSA: methicillin-resistant *S. aureus*; ^∗^^∗^meth: methicillin; oxa: oxacillin.

## Data Availability

The data that support the findings of this study are available. Anyone interested can get upon reasonable request from the corresponding author.
